# Barriers and Facilitators to Physical Activity Among Older Adults in Residential Aged Care Facilities: A Mixed Methods Systematic Review Using the Social Ecological Model

**DOI:** 10.1177/08982643241302209

**Published:** 2024-11-22

**Authors:** Sumana Baidya, Cath J. Connolly, Jasmine M. Petersen, Claire Baldwin, Maayken E. L. van den Berg, Isobel Harris, Lucy K. Lewis

**Affiliations:** 1 Caring Futures Institute, College of Nursing and Health Sciences, 64767 Flinders University, Bedford Park, SA, Australia

**Keywords:** physical activity, exercise, assisted living, barriers, facilitators, PROSPERO CRD42018104818

## Abstract

**Objective:**

To ascertain the barriers and facilitators to physical activity (PA) for older adults in Residential Aged Care Facilities (RACFs), from the perspective of residents, staff, and family.

**Methods:**

A mixed-methods systematic review, underpinned by the Social Ecological Model (SEM). Five databases were searched from inception to May 2024. Data synthesis followed a convergent integrated approach, with relevant quantitative data ‘qualitised’ and synthesised with qualitative data using thematic synthesis.

**Results:**

This review included 67 studies (40 qualitative, 16 mixed-methods, and 11 quantitative). Thematic synthesis identified 51 key themes (27 facilitators and 24 barriers), spanning all levels of the SEM. Intrapersonal factors (e.g. poor general health) were the most commonly cited barriers (*n* = 53 studies), and interpersonal factors (e.g. social support) the most commonly reported facilitators (*n* = 55 studies) to PA in RACFs.

**Discussion:**

An interplay of multi-level factors must be addressed in the development and implementation of strategies to promote PA in RACFs.

## Introduction

Population growth and increasing life expectancy will result in the number of people aged 65 years and older more than doubling from 703 million in 2019, to over 1.5 billion by 2050, placing increased demand on health and social services globally (United Nations, 2020; WHO, 2023). In 2019, approximately 11% of people aged 65 and over were receiving long term care, delivered either at home or in a Residential Aged Care Facility (RACF) (OECD, 2021). Despite the international shift in focus for older adults to stay home as long as possible, residential care remains prominent, with only minimal increases in the proportion of long-term care recipients receiving home-based care globally, from 67% in 2009 to 68% in 2019 (OECD, 2021; WHO, 2023). While international life expectancy has increased (United Nations, 2020), health-adjusted life expectancy has not kept pace (WHO, 2023). Improving the number of years lived in good health is an international priority, with healthy lifestyle behaviours such as physical activity (PA) ideally placed to contribute to this objective (Dipietro et al., 2019; Kaufman et al., 2023).

Physical activity, defined as ‘*any bodily movement produced by skeletal muscles that requires energy expenditure’* is a major contributor to successful and healthy ageing (Bauman et al., 2016; WHO, 2022). As age increases, PA decreases, increasing the risk of chronic diseases and disability, with negative consequences to health and social systems, economic development, community well-being, and quality of life (AIHW, 2018; Gomes et al., 2017; Sun et al., 2013; van Ballegooijen et al., 2019; Watson et al., 2016; WHO, 2020). There is clear evidence supporting the benefits of PA for older adults in improving functional ability (physical, social, cognitive, and emotional domains), maintaining independence, preventing falls, reducing and managing chronic conditions, and increasing quality of life (Bauman et al., 2016; Moreno-Agostino et al., 2020; Pinheiro et al., 2022; WHO, 2020, 2022). The benefits of PA extend to older adults living in RACF, with specific improvements for this population noted in functional mobility, autonomy, anxiety levels, balance, social interactions, and quality of life (Baldelli et al., 2021).

The most recent *Guidelines on Physical Activity and Sedentary Behaviour* recommend older adults (65+) with (and without) chronic conditions engage in 150–300 minutes of moderate-intensity, or 75–150 minutes of vigorous-intensity, aerobic PA, and incorporate multicomponent PA that has an emphasis on functional balance and strength training, three or more days a week (WHO, 2020). Despite clear guidance, PA levels among older adults remain low (AIHW, 2022). This pattern is observed internationally, with approximately 75% of older Australians not meeting PA guidelines (AIHW, 2018), approximately 30% of older Americans not engaging in any PA at all (Watson et al., 2016), and older adults in the Netherlands spending up to 80% of their waking time sedentary (Koolhaas et al., 2017). Although there are no widely accepted PA guidelines for the RACF setting, it is recognised that some PA is better than none (WHO, 2020). There is a paucity of research investigating activity behaviours of older adults specifically in residential settings. Of the few studies that have been reported, PA levels of residents appear very low, both in terms of volume and intensity of activity. Cognitively intact older adults in RACF have been shown to engage in less than 5 minutes of moderate to vigorous PA per day and spend up to 85% of their waking time sedentary, with daily sedentary time higher among residents in high-level, compared with intermediate-level care (11.6 vs. 9.5h/day, respectively) (Leung et al., 2021; Lewis & Lange, 2019; Mc Ardle et al., 2021; Parry et al., 2019; Reid et al., 2013).

The average length of stay in RACF has declined in the last 10 years and is currently between 23 to 31 months in Australia, with differences by age, sex, and marital status (Zhang et al., 2023). While this average length of stay is projected to continue to decline to 2040 due to an increased focus on aged care in the home, it is an ongoing priority for residents to maintain activity levels to maximise physical and mental health, social connections, and quality of life (Zhang et al., 2023). It is therefore crucial to identify the factors that impact RACF residents’ activity levels (Douma et al., 2017; Narsakka et al., 2022). Existing research suggests that the PA of older adults in RACFs is facilitated (or undermined) by many multi-level factors. For example, identified facilitators include accessible and safe facilities, supportive professionals, integration with the community, family and volunteers, and adequate activities to socialise and be active (Narsakka et al., 2022). On the other hand, compatibility of the environment with resident ability, equipment, and the accessibility, security, comfort, and aesthetics of the environment, have been cited as barriers (Douma et al., 2017). However, the barriers and facilitators of PA in RACFs have not been previously systematically reviewed or synthesised. The Social Ecological Model (SEM) (Bronfenbrenner (1977a) is a framework that acknowledges that human behaviour (e.g. PA) is influenced by an interplay of intrapersonal, interpersonal, organisational, environmental, and policy factors. Application of the SEM to ascertain multi-level influences of PA among older adults in RACFs should provide valuable insight, to guide the development and implementation of approaches that are most likely affect change in activity behaviours.

This review, underpinned by SEM, aimed to systematically identify and synthesise the evidence on the perceived barriers and facilitators to PA for older adults living in RACF from the perspectives of all stakeholders. An understanding of the barriers and facilitators to PA, categorised within the SEM will inform the development and implementation of targeted interventions that are acceptable to older adults, their families, staff, policymakers, and other stakeholders (Bronfenbrenner, 1977a).

## Methods

This review was conducted in accordance with Johanna Briggs Institute (JBI) methodology for mixed methods systematic reviews and reported using the Preferred Reporting Items for Systematic Reviews (PRISMA) (Lizarondo et al., 2020b; Page et al., 2021) (see Additional File 1). The protocol was prospectively registered (PROSPERO CRD42023423037) (Baidya et al., 2023; Shamseer et al., 2015).

### Search Strategy

The initial search strategy was developed a *priori*, peer reviewed by an academic librarian and adapted for each database (MEDLINE, EMBASE, CINAHL, CENTRAL The Cochrane Library, and AgeLine). An example search strategy for Medline is provided in Box 1. Wherever possible, both subject heading and keyword searches were used, relating to older adults, aged care, PA, barriers, and facilitators. There were no limits on publication date and only English studies were included. The final search was undertaken in May 2024. Reference lists of all included studies were searched, and forward citation searching of included studies was completed.

**Box 1:** Search Strategy (Medline)

1. ‘Aged, 80 and over’/or Aged/
2. Ageing/
3. Geriatrics/
4. (Elder^^*^^ or geriatric^^*^^ or gerontolog^^*^^ or old age^*^ or senior^^*^^ or late^^*^^ life).tw, kf.
5. ((Old^*^ or age^*^ or ageing) adj1 (person or people^*^ or adult^*^ or resident^*^ or population^*^ or men^*^ or women^*^ or male^*^ or female^*^)).tw, kf.
6. (Aged adj1 (‘65’ or ‘70’ or ‘75’ or ‘80’ or ‘85’)).tw, kf.
7. Or/1–6
8. Homes for the aged/or residential facilities/or assisted living facilities/or long-term care/
9. Institutionalization/
10. (Residential care or aged care or nursing home^*^ or residential facilit^*^ or assisted living or care home^*^ or old age home^*^ or long-term care or long term care or institution^*^ or extended care facilit^*^ or home^*^ for the aged).tw,kf.
11. Or/8–10
12. Exercise/or exercise therapy/
13. walking/or warm-up exercise/
14. (Exercise^*^ or physical^*^ activ^*^ or walk^*^ or restorative care or reablement or re-ablement or rehabilitat^*^ or reactivation program or re-activation program or strength^*^ train^*^ or resistance train^*^ or functional train^*^ or strength^*^ program^*^ or resistance program^*^ or functional program^*^ or physiotherapy or physical therap^*^ or occupational therap^*^).tw, kf.
15. 12 or 13 or 14
16. Attitude to health/or attitude/
17. (Barrier^*^ or hinder or facilitat^*^ or motivat^*^ or encourage or support^*^ or determinant or preference^*^ or knowledge or attitude^*^ or belief^*^ or perception^*^ or enabl^*^ or culture).tw, kf.
18. Or/16–17
19. 7 and 11 and 15 and 18
20. (Note or editorial or comment or letter or news).pt.
21. 19 not 20
22. Limit 21 to English language

### Eligibility Criteria

Primary published studies were included with original quantitative and/or qualitative data on the barriers or facilitators to PA for older adults in RACF from the perspective of older adults (65+ years), family, staff, or other stakeholders. For this review, RACF was defined as an institutional setting where care is provided for older people 24 hours a day, seven days a week, and assistance is provided for activities of daily living (ADL). While there are many different terms used for RACF internationally, this definition is consistent with an international consensus definition of ‘nursing home’ (Sanford et al., 2015). Studies with participants residing in medical or hospice facilities or the community were excluded. Conference abstracts, proceedings, and dissertations were excluded.

### Study Selection

Citations were uploaded into Endnote, duplicates removed, and exported into Covidence for screening (Covidence systematic review software, 2023; EndNote, 2023). Titles and abstracts were screened by two independent reviewers against eligibility criteria. Studies that met the inclusion criteria, or where ambiguity existed, were retrieved in full and screened by two independent reviewers. Disagreements were resolved through discussion, with a third independent reviewer as required.

### Assessment of Methodological Quality

Included studies with eligible data were critically appraised by two independent reviewers using the *Analytical Cross Sectional Studies* and *Qualitative Research* JBI checklists (Lockwood et al., 2015; Moola et al., 2020). If disagreements were unable to be resolved, a third reviewer was consulted. Studies were not excluded based on their methodological quality.

### Data Extraction and Transformation

Relevant data were extracted by one reviewer and cross-checked by a second reviewer, using standardised JBI data extraction methodology (Lizarondo et al., 2020a; Microsoft Corporation, 2018). Data were extracted including the population, methods, context, and barriers and facilitators to PA. Qualitative data were extracted verbatim, as well as relevant themes and subthemes. Relevant quantitative data were ‘qualitised’, by transforming data into textual descriptions to answer the review question by repeated detailed examination (Lizarondo et al., 2020b).

### Data Synthesis and Integration

Data synthesis followed a convergent and integrated approach, assembling the ‘qualitised’ and qualitative data (Lizarondo et al., 2020b). Assembled data were categorised and pooled based on similarity in meaning to produce a set of integrated findings, using reflexive thematic analysis and synthesis (Braun & Clarke, 2019). Two reviewers familiarised themselves with the assembled data, with codes inductively emerging. Codes were then deductively allocated in the SEM levels of Intrapersonal, Interpersonal, Organisational, Environmental, and Policy and themes constructed (Bronfenbrenner (1977a). Potential themes were first reviewed, defined, and named by the two reviewers, and subsequently reviewed by the research team.

### Certainty of Evidence

Assessment of the certainty of the evidence (e.g. using GRADE or ConQual) is currently not recommended for mixed methods systematic reviews using the integrated approach (Lizarondo et al., 2020b). Therefore, results were presented using a schematic diagram, accompanied by narrative explanation of themes and integrated findings.

## Results

### Study Selection

**Figure 1.**
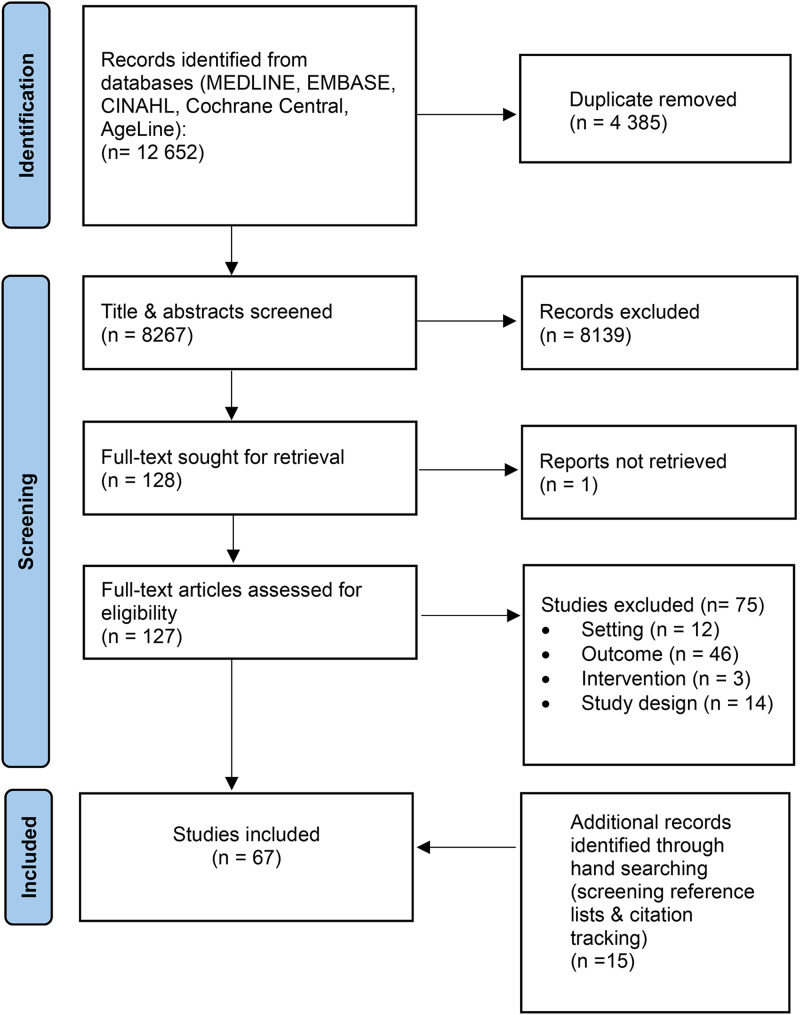
PRISMA flow diagram.

After removal of duplicates, the e-database search yielded 8267 citations. Following title and abstract screening, 127 papers underwent full-text review, of which 52 were eligible (Figure 1). An additional 15 eligible studies were identified through forward citation tracking and checking of reference lists, resulting in 67 included studies.

### Characteristics of Included Studies

Characteristics of the studies are presented in Additional File 2. Included studies (*n* = 67) were published between 1987 and 2024, with over half (50.7%, *n* = 34) published in the past five years. Studies were conducted in Europe (*n* = 18), United States (*n* = 14), Australia (*n* = 13), United Kingdom (*n* = 7), Canada (*n* = 4), Taiwan (*n* = 3), China (*n* = 2), South Africa (*n* = 2), Brazil (*n* = 1), India (*n* = 1), Japan (*n* = 1), and New Zealand (*n* = 1). Forty studies used qualitative data collection methods, followed by mixed methods (*n* = 16; including *n* = 1 quasi-experimental study), and quantitative (*n* = 11).

### Participants and Setting

The total sample size across included studies was 3593 participants, with sample sizes ranging from 4 to 390 participants. Most studies included RACF residents (*n* = 48). Samples also incorporated various other stakeholders including facility staff (*n* = 25), physiotherapists (*n* = 11), nurses (*n* = 9), and family (*n* = 10). While all studies met the criteria for RACF, there were many different terms and descriptions for the setting (e.g. assisted living, nursing home etc.) (Additional File 2).

### Interventions

Thirty studies (44.7%) reported perceived barriers or facilitators in relation to a PA intervention. Interventions most commonly included multi-component exercise (*n* = 10), e- or m-health (e.g. app, virtual reality; *n* = 4), non-specific physical exercise (*n* = 3), dancing (*n* = 2), high-intensity exercise (*n* = 2), multifaceted walking (*n* = 2), and sit-stand exercise (*n* = 2). Other interventions (*n* = 1 each) comprised postural-stability instruction, physiotherapy, Tai-chi/Yoga, progressive resistance training, and combined exercise with bingo.

### Schematic of the Synthesis

**Figure 2.**
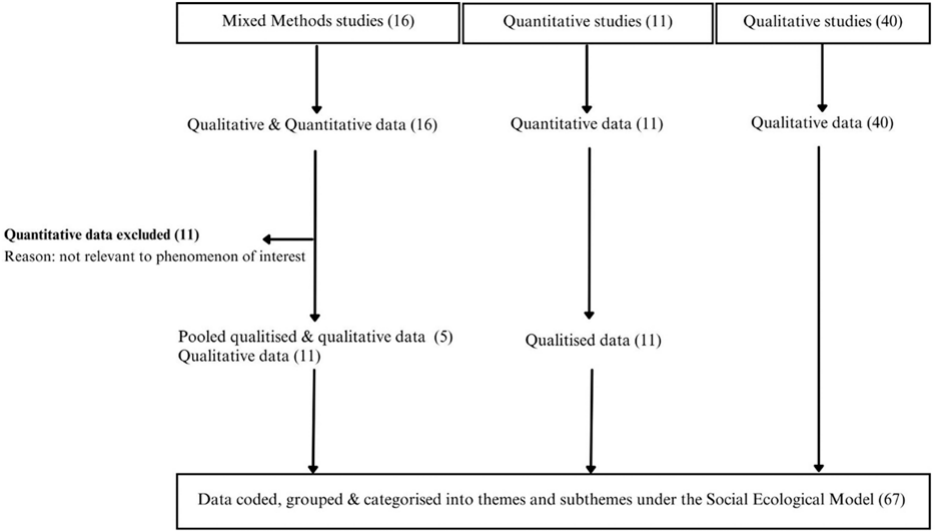
Schematic of synthesis.

The final data synthesis included qualitative data from 40 qualitative studies and 11 mixed methods studies, qualitised data from 11 quantitative studies (see Additional File 3 for examples of qualitised data), and pooled data from five mixed-methods studies. Quantitative data from 11 of the mixed-methods studies (*n* = 16) did not report on the phenomena of interest and were therefore excluded (see Figure 2).

### Barriers and Facilitators to PA

Twenty-four themes pertaining to PA barriers and 27 facilitator themes were identified. Each of these themes (and sub-themes) has been mapped to the SEM (Tables 1 and 2). Themes are presented in italics in the text.

**Table 1.** Barriers to Physical Activity (PA) for Older Adults in Residential Aged Care Facilities (RACF), From the Perspective of Residents, Staff, and Others, Under the 5 Levels of the Social Ecological Model (SEM).

SEM Level	Theme, Subtheme	Resident	Staff	Other
Intrapersonal	**Dependence & lack of autonomy**			
	Requires physical assistance	20, 35, 44, 63	22, 29, 52, 59	RO: 35
	Resident lack of autonomy & choice regarding PA	5, 22, 26, 60, 61, 62, 41	3, 7, 22, 34, 52, 59, 66, 41	F: 7
	**Negative emotions & feeling**	13, 19, 44	44	
	Self-conscious	15, 19, 20, 30, 40, 65	22	
	Fear	5, 13, 15, 16, 19, 30, 31, 33, 39, 44, 48, 56, 62	7, 22, 52, 59	
	Negative feelings about aged care	19, 35	22	
	Loneliness & isolation	26	20, 22	
	Grief & loss	26	22	
	**Poor general health**	1, 5, 15, 22, 31, 39, 48, 54, 63, 27, 64, 36	27, 64, 37	F 27, 37; V: 64
	Co-morbidities	13, 15, 26, 30, 39, 44, 45	3, 18, 21, 22	
	Health history	22, 44, 56, 62		
	Illness	30, 44	2	
	Injury	30, 13		
	**Negative attitudes**	1, 15, 20, 32, 48, 50,56, 62	2, 3, 20, 29, 38	
	Negative beliefs	15, 19, 20, 22, 26, 39, 48, 49, 50, 53, 62	22, 29, 52	
	Poor self-efficacy	56, 62	35, 52	PM: 34
	Lack of motivation	15, 19, 20, 22, 39, 44, 48, 62, 12, 64, 42	22, 12, 42	V: 64
	**Previous exposure to PA**			
	Lack of, or negative past experience	5, 15, 26, 44	22	
	Lack of knowledge about PA	1, 15, 22, 44, 62	2, 10	
	**Risk & safety**	27	27	F: 27
	Importance of safety	44, 37	9, 37	F: 20, 37 PM: 20
	Risk of harm	15, 16, 49, 62	2, 3, 16, 21, 66, 9	
	Contraindications		2	
	**Limitations**	15, 22, 44, 12, 42	22, 29, 38, 12, 42, 23	
	Sensory limitations	5, 13, 15, 19, 20, 22, 25, 26, 30, 31, 44, 45, 48, 53, 61, 62	2, 3, 18, 21, 22, 52, 66	RO: 35
	Physical limitations	15, 19, 30, 35, 39, 44, 53, 62	2, 3, 6, 16, 21, 22, 59	
	Functional limitations	5, 13, 15, 20, 22, 26, 30, 31, 32, 48, 39, 62	22, 59, 52	PM: 7
	Cognitive limitations	5, 13, 19, 31, 61, 62, 63	21, 22, 52, 59	
	Responsive behaviours		3, 11, 17, 18, 29, 52	
Interpersonal	**Influence of individual staff**			
	Staff lack of motivation	5	2, 3, 38	
	Staff own well-being	5	3	
	Staff fearful	5	3, 18	
	Staff lack of knowledge & experience	5	3, 52, 59	
	Negative staff perceptions		3, 18, 29	
	**Family lack of knowledge about PA**		3, 52	
	**Lack of support for residents**	19	22	
	Lack of encouragement	1, 16, 19, 20, 62		F: 7
	Lack of family support		46	
	**Difficulty engaging with others**			
	Dislike of group setting	15, 19, 35, 65	2, 22, 51	F: 17
	Lack of, or difficult relationships	5, 19, 26, 30, 39, 65	3	
	Poor communication	19, 34	18, 22	
Organisational	**Organisational priorities**		29, 10	
	Inadequate staff support	35	3, 6, 7, 22, 29	
	Inadequate exercise opportunities	5, 19, 20, 62, 36	2, 41	
	Poor staff culture		11, 38, 59	
	Care prioritised over PA	27	2, 3, 18, 29, 52, 8, 27	RO: 35, F: 27
	**Care approach**			
	Approach facilitates dependence		2	
	Staff use of physical restraint		66	
	Staff overuse of wheelchairs		7, 52	RO: 35
	Cannot force resident to participate		2, 3, 59, 66	F: 7
	**Organisation & structure of PA**		28	
	Inappropriate time of day	22, 24	6, 22	F: 7
	Poor flexibility & adaptability of PA	5, 34		
	Type or duration of PA undesirable	48, 61, 62	2	F:17
	PA not tailored to resident capabilities	5, 48, 62		
	**Lack of, or poor information sharing about PA**	48, 53, 62		
	Residents unaware of resources & opportunities available	5, 22, 34, 48		
	**Heterogeneity of resident abilities**	62	2	
	**Inadequate resources**	12, 42	3, 11, 29, 12, 42, 10	
	Inadequate funding		2, 3, 6, 7, 46, 8, 10	
	Workload management & competing demands	63, 12, 4	2, 3, 6, 7, 11, 17, 29, 46, 52, 59, 66, 12, 10, 4	F: 17, RO: 34
	Workforce issues	5, 44, 27, 12, 41, 4	2, 3, 6, 7, 29, 35, 38, 59, 66, 9, 27, 12, 41, 10, 4	RO: 35, F: 27
Environmental	**Unable to access facilities independently**		22	RO: 34
	Transport to/from PA limiting access	33	2, 6, 7, 34	RO: 34
	Doorway entrance limiting access	34	6, 7, 34, 35	RO: 34
	Not accessible with gait aids or wheelchairs	34	34	RO: 34
	**Lacking connection to community**		2, 20	
	**Resident experience of the environment**			
	Poor weather	44, 27, 64	6, 27	F: 27; V: 64
	Inappropriate music	5, 19		
	Poor visual experience	5		RO: 35
	**Safety**	33		
	Uncertain about neighbourhood safety	62		
	Environmental safety hazards	62	6, 34	
	**Lack of, or poor infrastructure**	1, 5, 19, 33, 44, 27, 64, 47, 36	2, 3, 6, 7, 11, 34, 27, 37	RO: 34; F: 27, V: 64
Policy	**Rules & regulations**	62	20, 29, 10	
	Not allowed to leave the facility	26		
	Hygiene regulations		20	
	Safety regulations	27	20, 27	F: 27
	**Policies deter interest**	44		

*Note. *F Family, PM Policy Maker, RO Research Officer, V Volunteer; **[1]** Aro et al. (2018); **[2]** Baert et al. (2016); **[3]** Baert et al. (2015); **[5]** Bender et al. (2021); **[7]** Benjamin et al. (2011); **[8]** Binns et al. (2023); **[9]** Bradwell et al. (2023); **[10]** Brett et al. (2023); **[11]** Brett et al. (2018); **[12]** Brusco et al. (2024); **[13]** Chao et al. (2016); **[15]** Chen (2010); **[16]** Chu et al. (2021); **[17]** Chu et al. (2022); **[18]** Galik et al. (2009); **[19]** Gebhard and Mir (2021); **[20]** Giné-Garriga et al. (2019); **[21]** Gomaa et al. (2020); **[22]** Guerin et al. (2008); **[23]** Högstedt (2023); **[24]** Hummer et al. (2015); **[25]** Ingrid and Marsella (2008); **[26]** Jeon et al. (2019); **[27]** Jepson et al. (2023); **[28]** *Krafft et al., 2023; **[29]** Kuk et al. (2018); **[30]** Lindelöf et al. (2012); **[31]** Linhares et al. (2022); **[32]** Liu and Hu (2015); **[33]** Lu et al. (2011); **[34]** Mahrs Träff et al. (2020); **[35]** Mahrs Träff et al. (2019); [**36]** Meyer, Golenko, Cyarto, O'Keefe, Bonney, et al. (2023); **[37]** Meyer, Golenko, Cyarto, O'Keefe, Cooley, et al. (2023); **[38]** Mihalko and Wickley (2003); **[39]** Moran et al. (2015); **[40]** Narkauskaitė-Nedzinskienė et al. (2020); **[41]** Narsakka et al. (2023); **[42]** Ofosu et al. (2023); **[44]** Phillips and Flesner (2013); **[45]** Pienaar et al. (2004); **[46]** Post et al. (2020); **[47]** Poveda-López et al. (2023); **[48]** Poveda-López et al. (2022); **[49]** Prevc and Doupona Topic (2009); **[50]** Rapp and Carlson (1987); **[51]** Raynor et al. (2020); **[52]** Resnick et al. (2008); **[53]** Rohisha et al. (2017); **[54]** Ruuskanen and Parkatti (1994); **[56]** Stathi and Simey (2007); **[59]** Turpie et al. (2017); **[60]** Vos et al. (2019); **[61]** Wang et al. (2021); **[62]** Webster et al. (2023); **[63]** Weeks et al. (2008); **[64]** Weissbach et al. (2023); **[65]** Wootten et al. (2022); **[66]** Wu et al. (2013).

**Table 2.** Facilitators to Physical Activity (PA) for Older Adults in Residential Aged Care Facilities (RACF), From the Perspective of Residents, Staff, and Others, Under the 5 Levels of the Social Ecological Model (SEM).

SEM	Theme, Subtheme	Residents	Staff	Other
Intrapersonal	**PA provides purpose & meaning to life in aged care**	14, 19, 40, 43, 48, 49, 60, 61	2, 3	
	PA adds value to daily routine	19, 34, 44, 56, 60, 61, 65	2, 59	
	PA is a good use of time	31, 60	2	
	Need for self-expression	40		
	**Independence & autonomy**	5, 20, 51, 65	18	
	PA for independence	20, 22, 26, 30, 34, 35, 40, 43, 51, 56, 60, 62, 65	2, 3, 11, 22, 52	F: 20, O: 35
	PA to manage ADLs	19, 20, 26, 30, 31, 40, 43, 48, 51	17, 51	F: 46
	Resident choice regarding PA participation & type	20, 43, 44, 48, 60, 62, 63	2	
	PA to avoid dependence	14, 20, 26, 30, 48, 60, 65	11, 51	
	**Positive attitudes**	14, 20, 26, 30, 31, 34, 35, 40, 43, 44, 58, 60, 62, 63	2, 1, 51	F: 51
	Motivated to participate in PA	13, 14, 19, 26, 30, 31, 34, 43, 48, 56, 60, 64, 4	2, 3, 22, 4	F: 20, V: 64
	Realistic expectations of PA & personal capabilities	31, 40, 55, 56, 61		
	**Positive feelings**	13, 30, 34, 43, 44, 55, 56, 60, 61, 4	52, 4	F: 17, 51
	Feeling unafraid	30, 56		
	Promotes enjoyment, fun, & happiness	13, 16, 19, 20, 30, 31, 43, 44, 48, 53, 55, 56, 58	2, 7, 11, 18, 22, 46, 52, 66	F: 51
	Prevents negative emotions, feelings, & moods	5, 14, 19, 22, 33, 48, 61, 62, 65	2, 3	F: 51
	Increases optimism	13, 14, 30, 43, 60		
	Creates a sense of pride	20, 44		F: 17
	Creates a sense of achievement	13, 31, 43, 55, 56, 58	2	
	**Self-awareness**	40		
	Self-efficacy	14, 30, 43, 55, 56, 60, 62, 4	3, 46, 4	F: 17, 46
	Improves self-image/self-esteem	40, 43, 53	2	
	Fear of loss of dignity	14		
	**Resident capabilities impact participation in PA**	19, 40		
	Impact of cognitive abilities on participation	30	3, 6, 22	
	Impact of physical abilities and general wellness on participation	5, 7, 30	6	
	**Past experience & knowledge about PA**			
	Positive past experiences of PA	2, 26, 44, 48, 61	3, 22	
	PA previously important to resident	5, 14, 19	22	F: 46
	Resident knowledgeable about PA	1	2, 22	
	Resident provided opportunities to increase PA knowledge	30, 58, 61		
	**Avoiding sedentary behaviour**			
	Avoid sitting	14, 60, 63, 65		
	Risks &negative consequences of inactivity	30	2, 3	
	**Benefits of PA**	30, 31, 48, 51, 56, 58, 61, 64, 36	2, 52, 8, 37	F: 11, F: 37, V: 64
	General health & wellbeing benefits	13, 16, 19, 20, 26, 30, 32, 40, 44, 48, 50, 53, 55, 56, 60, 61, 65	2, 3, 38	
	Sensory benefits	5, 19, 30, 48, 55, 62	3	
	Communication benefits			F: 17
	Physical health benefits	13, 14, 22, 30, 33, 34, 40, 44, 53, 56, 60, 61	2, 3, 46, 66	
	Physiological benefits	19, 53, 61	51	
	Functional benefits	5, 13, 14, 19, 20, 30, 31, 32, 43, 44, 48, 51, 56, 60	2, 3, 11, 17, 22, 46, 51	F: 11, 48
	Psychological benefits	13, 14, 30, 31, 32, 43, 44, 48, 50, 53, 55, 60, 61, 62	2, 3, 11, 46, 51, 55, 66	F: 17, 46, 51
	Cognitive benefits	13, 16, 26, 30, 44, 48, 56, 61		F: 17
	Behavioural benefits		11, 46	
	Spiritual benefits	30, 61		
	Health prevention benefits	13, 14, 30, 32, 63	66	
Interpersonal	**Influence of individual staff**			
	Staff expertise	5, 48, 57, 36, 37, 4	3, 52, 67, 36, 37, 10, 23, 4	F: 36, 37; V: 36, 37
	Staff engagement	50, 56, 4	2, 3, 21, 46, 51, 52, 59, 9, 4	
	Staff views & beliefs		59, 8, 23	
	Instructor persona	5,30, 44, 50	18, 21, 52, 66	F:17
	Staff communication strategies & interpersonal skills		18, 21, 66	
	**Reduces burden on others**	14, 63	2, 3	
	Makes residents easier to live with	50		
	Don’t want to burden family	58		
	Easier to provide care		46, 52	
	**Psychological benefits extend to family**		2, 3, 52	F:20, 51
	**Resident relationship with staff & students**			
	Positive relationships facilitate participation	43, 51, 58	2, 7, 51	F: 51
	Trust & belief		3, 7, 18, 66	
	**Sense of accountability to others**	40		
	Adherence to support staff efforts	61, 43		
	Resident sense of commitment to program	13		
	Perceived obligation to exercise	58		
	**Social benefits**	13, 49, 65, 48, 1, 34, 44, 32, 55, 60, 61	2, 11, 16, 17, 22, 46, 51, 66	F:17
	PA facilitates interaction, connection, fun, enjoyment, and stimulation with others	5, 13, 20, 22, 26, 30, 34, 40, 51, 56, 58, 60, 61	3, 11, 16, 22, 51	F: 17, 46
	PA in a group setting	25, 30, 31, 48, 49, 62	2, 3	RO: 35
	**Support & encouragement**	20, 39, 44, 63, 12, 64, 42	12, 42	V: 64
	Support & encouragement of residents	1, 5, 20, 22, 25, 26, 34, 44, 48, 50, 52, 53, 58, 59, 61, 62, 63	2, 3, 6, 21, 22, 51, 59, 66	F: 2, 7, 20, RO: 35
	Staff support, cooperation, and teamwork	18, 36, 4	2, 3, 18, 22, 46, 52, 37, 4	F: 36, 4, V: 36, 4
	Interprofessional communication and collaboration	12, 36	12, 36, 10, 23	F: 36, V: 36
Organisational	**Appropriate resources & materials**		3, 18, 23	RO: 2
	Staffing	1, 56	3, 6, 18, 38	RO: 35
	Space			RO: 35
	Finances	36	2, 36	PM: 20, F: 36, V: 36
	**Program evidence**			
	Evaluation and implementation	4	8, 4	
	**PA embedded in facility strategic vision**		2, 3	
	PA contributes to positive facility image		2	
	Promotion of the PA program	36	36	F: 36, V: 36
	**Structure & approach of the PA service**	12	9, 12	
	Free PA service		2, 3	
	Scheduling	48, 65	18	F:17
	Organisation of PA program	5, 13, 44	2, 3, 52	
	Approach used by staff	26, 39, 44, 62	51, 52, 59, 66	F: 17, 51, RO: 35
	**PA options offered**			
	Appropriate intensity	40, 55, 61, 62		
	Integration of PA with usual care	12, 57, 42	12, 67, 42	
	Suitable exercises for resident capabilities	30, 31,44, 58, 61, 62, 12	12, 67, 23	F: 16
	Variation in PA options	14, 16, 19, 26, 34, 40, 44, 48, 55, 58, 62, 57, 36, 37	2, 16, 59, 66, 36, 37	F: 36, 37, V: 36, 37
	Other leisure activities that stimulate movement	21	7, 18	
Environmental	**Infrastructure that facilitates walking**	41, 36	2, 3, 6, 41, 36, 23	RO: 35, F: 36, V: 36
	Adequate space	35, 59, 62	59	PM: 20
	Outdoor space	35, 44	6, 35	
	Corridors	33, 48	6, 35	RO: 35
	Exercise facilities	1	2, 59	
	**Safety of environment**	33		
	Seating in corridors	33, 39		RO: 35
	Assistive devices	33	6, 18, 35	RO: 35
	Safe walking surfaces	33	6	
	**Resident experience of the environment**			
	Sensory experience	5, 16, 39, 55	18, 21	F: 20
	Nice weather		6, 20, 40, 44	
	**Community integration**	20, 26, 39	2, 20	F: 20
	**Accessible spaces**			
	Location of space	5, 33		
	Accessibility of outdoors	39	6, 35	
	Accessibility of exercise resources** **and equipment	26, 44, 48, 57, 36	36	RO: 35; F: 36, V: 36
Policy	**Policies**	5		
	Mandatory requirement for some residents to participate	5		

*Note. *F Family, O Observation, PM Policy Maker, RO Research Officer, V Volunteer; **[4]** Barrett et al. (2023); **[6]** Benjamin et al. (2009); **[14]** Chen and Li (2014); **[43]** Olsen et al. (2015); **[55]** Saravanakumar et al. (2018); **[57]** Swales et al. (2024); **[58]** Taylor et al. (2020); **[67]** Yokogawa et al. (2023).

### Intrapersonal

At the intrapersonal level, 53 studies (79.1%) reported barriers to PA for older adults in RACFs. Limitations (*n* = 38 studies) were the most prevalent (barrier) theme at the intrapersonal level, and across all levels of the SEM. Frequently cited barriers by both residents and staff pertained to sensory (e.g. dislike of touch or noise), physical (e.g. poor strength or balance), functional (e.g. mobility, ADLs) and cognitive limitations (e.g. poor memory, confusion). For example, a resident cited a barrier relating to sensory limitations ‘…I began to stop attending the sessions because I could not see the ball when it was coming. The ball would fall on the ground, and I began to get sad … I realised I was going blind’ (Linhares et al., 2022). Negative attitudes (*n* = 24; lack of motivation, negative beliefs), Poor general health (*n* = 23; e.g. co-morbidities, health history), and Negative emotions and feelings (*n* = 22; e.g. fear, self-consciousness) were also prominent barriers (particularly for residents) at the intrapersonal level (see Table 1).

Forty-seven studies (70.1%) cited facilitators to PA in RACFs at the intrapersonal level (see Table 2). Most of these studies (*n* = 39, 83.0%) reported facilitators pertaining to Benefits of physical activity. More specifically, general health and wellbeing (e.g. improved sleep and quality of life), functional (e.g. improved mobility), psychological (e.g. improved mood) and cognitive benefits (e.g. improved memory, concentration) were commonly reported (particularly by residents) to facilitate engagement in PA. Many studies also identified that Positive feelings (*n* = 33, 70.2%) support PA engagement in RACFs. Engaging in PA was suggested to promote enjoyment, fun, and happiness, and assist in preventing negative emotions and feelings such as isolation, loneliness, boredom, and depression. For example, ‘I enjoy exercising with the Wii games, and I enjoy the entertainment with the exercise….it (Wii games) gives you a sense of accomplishment’ (Resident, Chao et al., 2016). Other frequently cited facilitators to PA at the intrapersonal level were Positive attitudes (*n* = 27; e.g. motivation, realistic expectations of PA) and Independence and autonomy (*n* = 25, e.g. PA for independence and to manage ADLs).

### Interpersonal

Twenty-four studies (35.8%) reported barriers at the interpersonal level. *Difficulty engaging with others* was a prominent theme (*n* = 14 studies). Specifically, lack of, or difficult relationships (e.g. conflict between residents), dislike of group settings (e.g. feeling self-conscious), and poor communication (e.g. between staff and residents*)* were commonly reported barriers by residents. As stated by a resident, *‘If you don’t fit in with the people doing the classes and activities, you may as well not bother turning up’* (Wootten et al., 2022). *Influence of individual staff* (*n* = 8; e.g. staff fearful or lacking motivation, knowledge, and experience) and *Lack of support for residents* (*n* = 8; e.g. lack of encouragement) were also key barriers to PA participation in RACFs at the interpersonal level (see Table 1).

Interpersonal factors were the most cited facilitators (*n* = 55; 82.0% of studies), across all levels of the SEM. *Support and encouragement* (*n* = 35) was the most commonly reported facilitator. This included support and encouragement for residents (from peers, family and staff; *n* = 25), along with staff support, cooperation and teamwork (*n* = 8), and interprofessional communication and collaboration (*n* = 4). For example, ‘*Many of the residents who do come have been encouraged to come by the physiotherapists’.* (Staff, Guerin et al., 2008). *Social benefits (n* = 31) were also commonly reported facilitators to PA participation by both residents and staff. Physical activity was recognised to facilitate interaction, connection, fun, enjoyment, and stimulation. Other facilitators at the interpersonal level included *Influence of individual staff* (e.g. staff expertise, engagement, views, and beliefs), *Resident relationship with staff and students* (e.g. trust and belief), and *Sense of accountability to others* (e.g. adherence to support staff efforts).

### Organisational

Thirty-five studies (52.2%) reported barriers at the organisational level. Over half of these studies (68.5%), cited *Inadequate resources* (*n* = 24) as a barrier, particularly for staff, to support PA participation in RACFs. For example, ‘*they fund, but they never fund enough. They expect that… from you and then they’ll say, we’ll give money, but it doesn’t cover* [costs for staffing]’ (Staff member, Benjamin et al., 2011). More specifically, workforce issues (*n* = 17; e.g. insufficient staff, high staff turnover), workload management and competing demands (*n* = 16; e.g. staff time constraints), and inadequate funding (*n* = 7) were identified to adversely influence PA participation. *Organisational priorities* (*n* = 21) were also widely cited to limit PA participation. This largely included barriers pertaining to inadequate exercise opportunities (*n* = 7) and prioritisation of care over PA (*n* = 8).

Organisational facilitators to PA in RACFs were documented by 42 studies (62.7%). Many studies (*n* = 26, 62.0%) cited the importance of *PA options offered* in facilitating participation. Variation in PA options was widely recognised to support PA participation (*n* = 17), along with suitable exercise for resident capabilities (*n* = 11), and integration of PA with usual care (*n* = 4). The *Structure and approach of the PA service (n* = 19*)* also emerged as important to supporting PA in RACFs. The provision of a free PA service that is organised and structured, anticipates possible challenges, is well advertised to residents, incorporates flexible scheduling, and is offered on a regular basis (including weekends) and at appropriate times, was suggested to facilitate PA participation. Fewer studies reported organisational facilitators pertaining to *PA embedded in a facility strategic vision (n* = 3*;* e.g. contributes to positive facility image) or *Program evidence* (*n* = 2; e.g. evaluation and implementation).

### Environmental

At the environmental level, approximately one-quarter of studies (*n* = 19) reported barriers to PA participation in RACFs. Most of these studies (*n* = 15, 79.0%) reported *Lack of (or poor) infrastructure* as a deterrent to PA. For example, limited space and poor configuration were identified as barriers, in addition to outdoor environments that were not conducive to PA (no gardens or small courtyards). Other barriers at the environmental level included *Unable to access facilities* (e.g. not wheelchair accessible), *Resident’s experience of the environment* (e.g. inappropriate music and poor visual experience) and *Safe*ty (e.g. environmental safety hazards; unsafe ramps, and uneven paths). For example, *‘One thing* that is *very frightening for people who are not very sure about their walking is to look at the highly polished floor’* (Resident, Lu et al., 2011).

Twenty-three studies (34.3%) documented facilitators to PA participation at the environmental level. Over half of these studies (56.5%), cited that *Infrastructure that facilitates walking* is important to supporting PA, and this was reported across, residents, staff, and other stakeholders (e.g. family, volunteers). Environments with adequate space for ADLs, exercise classes and walking (with or without gait aids), outdoor spaces (courtyards, gardens, and balconies), long and wide corridors, and well-equipped exercise facilities, were identified to promote PA participation. For example, *‘I think this is one of the better accommodations. The way the environment is developed or planned, I think it works well and there are long corridors, and you can walk around so there are good places to practice walking skills’* (Staff, Mahrs Träff et al., 2020). *Accessible PA spaces* (e.g. location of space; central, nearby, easy to find) and *Resident’s experience of the environment* (e.g. positive sensory experience; fresh air, pleasant fragrance, and scenery) were also reported to facilitate PA.

### Policy

Few studies reported barriers (*n* = 7, 10.4%) or facilitators (*n* = 1, 1.4%) at the policy level. *Rules and regulations*, and more specifically, restrictions on residents leaving the facility, and hygiene and safety regulations, were cited as barriers to PA participation. For example, *‘A lot of safety and hygiene regulations in long-term care facilities have been applied in the past years that make it difficult for residents to engage in certain household activities’* (Staff, Bender et al., 2021). *Policies*, such as those requiring physician permission or staff supervision, were suggested to deter interest in PA. On the other hand, one study (of residents) also reported that policies mandating participation in PA may be beneficial in facilitating PA in RACFs (Bender et al., 2021).

### Methodological Quality

Five studies achieved the maximum quality score across all JBI checklist criteria (Binns et al., 2023; Gebhard & Mir, 2021; Gomaa et al., 2020; Narsakka et al., 2023; Phillips & Flesner, 2013). A summary of the appraisal of included studies is presented in Table 3 and the appraisal scores for each study are presented in Additional File 2.

**Table 3.** Percentage of Included Studies Meeting Each Critical Appraisal Item From the Relevant JBI Checklist.

JBI Checklist	No. of Studies	% of Studies Meeting Each Item									
		Q1 (%)	Q2 (%)	Q3 (%)	Q4 (%)	Q5 (%)	Q6 (%)	Q7 (%)	Q8 (%)	Q9	Q10
Qualitative^a^	56	70	98	98	97	97	16	38	95	93%	97%
Analytical Cross-Sectional^b^	16	81	94	62	77	50	38	54	88	NA	NA

a) Q1: Is there congruity between the stated philosophical perspective and the research methodology? Q2: Is there congruity between the research methodology and the research question or objectives? Q3: Is there congruity between the research methodology and the methods used to collect data? Q4: Is there congruity between the research methodology and the representation and analysis of data? Q5: Is there congruity between the research methodology and the interpretation of results? Q6: Is there a statement locating the researcher culturally or theoretically? Q7: Is the influence of the researcher on the research, and vice-versa, addressed? Q8: Are participants, and their voices, adequately represented? Q9: Is the research ethical according to current criteria or, for recent studies, and is there evidence of ethical approval by an appropriate body? Q10: Do the conclusions drawn in the research report flow from the analysis, or interpretation, of the data?

b) Q1: Were the criteria for inclusion in the sample clearly defined? Q2: Were the study participants and the setting described in detail? Q3: Was the exposure measured in a valid and reliable way? Q4: Were objective, standard criteria used for measurement of the condition? Q5: Were confounding factors identified? Q6: Were strategies to deal with confounding factors stated? Q7: Were the outcomes measured in a valid and reliable way? Q8: Was appropriate statistical analysis used?

For the included studies with qualitative data (*n* = 56), items relating to location of the researcher culturally or theoretically (item 6) and the influence of the researcher on the research, and vice-versa (item 7) were addressed poorly, with only 16% and 38%, respectively, meeting these criteria. Item 1, assessing the congruity between the stated philosophical perspective and research methodology, was fulfilled by 70% of studies, and over 90% of studies met the quality criteria for all other items. For the included studies with relevant quantitative data (*n* = 16), items relating to the identification of, and strategies to address confounding factors (items 5 and 6), and the reliable and valid measurement of outcomes (item 7), were met by fewer than 55% of studies (see Table 3). Majority of studies (>80%) fulfilled criterion relating to clearly defined inclusion criterion (item 1), description of study subjects and setting (item 2), and appropriate statistical analysis (item 8).

## Discussion

This review is the first to systematically identify the barriers and facilitators to PA for older adults in RACF from the perspectives of a range of stakeholders (e.g. older adults, facility staff, and family), underpinned by the SEM. A large and rapidly growing body of evidence spanning decades (1987–2024) has examined such factors, with 67 studies included in this review and over half (50.7%) published in the past five years. This speaks to the ongoing recognition that physical inactivity in RACFs is a prominent concern. The findings of this review have important implications for continued efforts to improve PA participation among older adults in RACFs.

A plethora of barriers (*n* = 24) and facilitators (*n* = 27) were identified spanning all levels of the SEM, highlighting the complexity of supporting PA participation among older adults in RACFs. A recent review of PA interventions targeted at older people in care homes, does, however, suggest that such interventions rarely consider the multi-level factors influential in PA participation (Wylie et al., 2023). This may underpin the highly sedentary behaviour of older adults in RACFs (Leung et al., 2021; Parry et al., 2019), despite the prevalence of existing interventions developed to support PA in these settings (Lewis et al., 2021; Wylie et al., 2023). In the future, strategies targeted at promoting PA in RACFs should address the complex interplay of multi-level factors that influence PA, and could usefully be guided by theoretical frameworks such as the SEM.

Overall, intrapersonal barriers were the most commonly cited factors limiting PA participation among older adults in RACFs. More specifically, sensory, physical, functional, and cognitive limitations were frequently recognised by various stakeholders (e.g. residents, facility staff) as barriers to PA. Existing research in other older adult subpopulations, including community-dwelling and those with dementia in care homes, have similarly reported that health status is an important contributor to physical inactivity (Gebhard & Mir, 2021). Conversely, among the most commonly reported facilitators were those at the intrapersonal level, including perceived benefits of PA (e.g. general health and wellbeing benefits) and positive attitudes and feelings (e.g. motivation, enjoyment). This highlights that intrapersonal factors are highly influential in both supporting (and undermining) PA in RACFs, and are therefore, an important target for intervention. Tailoring PA offerings to intrapersonal factors (e.g. health status) and appropriate management of limiting factors such as pain, may be key to improving participation, and may foster enhanced PA enjoyment (or positive attitudes) among older adults in such settings.

Most studies (82%) documented facilitators at the interpersonal level, and in particular, social support and encouragement of residents were widely cited to promote PA in RACFs. This fits with a large body of extant research indicating that social support is key to facilitating engagement in PA, across the lifespan (Farren et al., 2017; Lindsay Smith et al., 2017). Importantly, social support is a core component of many behaviour change theories (Bandura, 1999; Bronfenbrenner, 1977b; Rosenstock et al., 1988), and has long been recognised as a modifiable determinant of PA (McNeill et al., 2006). Enhancing the provision of social support for PA among residents in RACFs may therefore be key to improving the activity levels of this subpopulation, especially given that this review indicates that social benefits (e.g. connectedness) are another important facilitator of PA. Walking sports, for example, is one such promising approach, shown to be both acceptable (and suitable) for older adults (Scott et al., 2024) and foster social connectedness (Mulvenna & Leslie-Walker, 2021). This review also suggests that the provision of support for RACF staff to facilitate PA is key. Ensuring that staff have the skills, knowledge, and experience to support PA participation and build therapeutic/caring relationships with residents, along with efforts to promote interprofessional communication and collaboration, will be beneficial to increasing PA in RACF settings. Taken together, this highlights that approaches to support PA in RACFs, that consider and target various stakeholder groups (e.g., residents, facility staff), will likely be of most value in affecting change in activity behaviours.

This review further indicates that organisational factors exert an important influence on PA participation in RACFs. Many studies, for example, reported that inadequate resources (e.g. workforce issues, competing demands, and inadequate funding) limit RACF staff capacity to support and prioritise PA participation. This is not surprising given the ongoing recognition of the systemic issues in the aged care sector (workforce shortages, insufficient funding, poor leadership), and calls for urgent action to address these issues to improve the quality of routine care (Xiao et al., 2021). Relatedly, in Australia, a Royal Commission into Aged Care Quality and Safety (which was targeted at driving reform), recommended that the needs and preferences of residents (and associated stakeholders; e.g. residents, families, and staff) must be at the forefront of high-quality (and safe) care provision (Royal Commission into Aged Care Quality and Safety, 2021). As identified in the present review, this must also extend to PA offerings in RACFs. This could, for example, include co-designing PA offerings with key stakeholder groups to ensure that such offerings are acceptable, appropriate, and suitable for all involved (Lewis et al., 2021).

Fewer than half (47.7%) of the included studies reported environmental facilitators or barriers to PA in RACFs. Lack of (or poor) infrastructure (e.g. limited space and poor configuration) and inaccessible facilities (e.g. location of space and limited access to exercise equipment) were cited to inhibit PA participation. Modifying existing environmental structures in RACFs may ensure that such environments support PA, and this is consistent with conclusions drawn from existing reviews of setting-related influences on PA in aged care (Douma et al., 2017; Narsakka et al., 2022; van den Berg et al., 2020). This could for instance, include enhancing the walkability of RACFs (long and wide corridors), adequate, well-equipped and safe spaces for exercise, and additional green spaces (parks, gardens, and court yards). It has been recognised that the built environment of RACFs is important to facilitating long-term PA participation by providing opportunities for activity that are integrated into daily life (Wylie et al., 2023). Ongoing efforts to ascertain design features of RACF environments that are most conducive to PA are therefore necessary.

Factors pertaining to policy were reported by 10% of studies. The settings-based approach to health promotion suggests that the macro-level (e.g. policies, guidelines) underpin the capacity of a setting to support health behaviours (Kokko et al., 2014). More specifically, macro (or policy) level factors are cited to exert a direct effect on the resources or supports (e.g. time, money, built environment, and stakeholder attitudes) available for settings-based health promotion (Kokko et al., 2014). Policy level factors are, however, rarely considered in strategies to support PA in RACFs (Wylie et al., 2023). They should therefore be a critical focus in future, and may drive changes to support PA participation that permeate the RACF setting (e.g. organizational and environmental levels).

### Implications

This review has important implications for improving PA participation among older adults in RACF settings. The findings highlight that promoting PA in RACFs is complex, with many barriers and facilitators, spanning all levels of the SEM. It is therefore, important that the development of strategies to improve PA in RACFs that address barriers and facilitators at the intrapersonal (e.g. limitations and perceived benefits of PA), interpersonal (e.g. social support and influence of staff), organisational (e.g. inadequate resources and PA offerings), environmental (e.g. poor infrastructure and accessible spaces), and policy levels (e.g. rules and regulations and mandatory requirements). Our findings further suggest that a one-size fits all approach is not appropriate in RACFs. Instead, strategies to support PA should be tailored (or adaptable) to factors across the SEM (e.g. intrapersonal factors; health status and limitations). Finally, considering all stakeholder groups (e.g. residents, family, and facility staff) in the development and implementation of strategies to support PA must be a priority. The utilisation of co-design methodology with these key stakeholder groups could usefully underpin efforts to improve PA among older adults in RACFs.

### Strengths and Limitations

This review has several strengths. It is the first to systematically identify the barriers and facilitators to PA for older adults in RACF from the perspectives of a range of stakeholders (e.g. older adults, facility staff, and family). The review was prospectively registered, followed PRISMA guidelines, and used a robust and rigorous methodology. It comprises a large body of qualitative and quantitative research (spanning decades), providing comprehensive insight into the factors that support and undermine PA participation in RACFs. There are, however, some limitations that should be acknowledged. We were not able to quantify the impact of the various multi-level factors, for example, environmental and policy factors were less frequently reported but may have large and widespread downstream influences on interpersonal and intrapersonal factors. This review was limited to peer-reviewed literature published in the English language, which resulted in the majority of included studies originating from high-income countries and may have introduced publication bias. It should also be acknowledged that just over half of the included studies were published in the last five years, and it is likely that the COVID-19 pandemic would have impacted the findings of studies collecting data in the RACF setting during this time.

### Conclusion

This review, underpinned by the SEM, provides critical insights into the facilitators and barriers to PA participation among older adults in RACFs. The findings highlight that a complex interplay of multi-level factors must be addressed in the development and implementation of strategies to promote PA in RACFs. These strategies should also be adaptable and consider the needs (and preferences) of all key stakeholders. This review is critical to driving ongoing efforts to improve PA participation among older adults in RACFs, a subpopulation that is highly sedentary and experiences poor health outcomes.

## Supplemental Material

Supplemental Material - Barriers and Facilitators to Physical Activity Among Older Adults in Residential Aged Care Facilities: A Mixed Methods Systematic Review Using the Social Ecological Model Supplemental Material for Barriers and Facilitators to Physical Activity Among Older Adults in Residential Aged Care Facilities: A Mixed Methods Systematic Review Using the Social Ecological Model by Sumana Baidya, Cath J. Evans, Jasmine M. Petersen, Claire Baldwin, Maayken E. L. van den Berg, Isobel Harris, and Lucy K. Lewis in Journal of Aging and Health

Supplemental Material - Barriers and Facilitators to Physical Activity Among Older Adults in Residential Aged Care Facilities: A Mixed Methods Systematic Review Using the Social Ecological Model Supplemental Material for Barriers and Facilitators to Physical Activity Among Older Adults in Residential Aged Care Facilities: A Mixed Methods Systematic Review Using the Social Ecological Model by Sumana Baidya, Cath J. Evans, Jasmine M. Petersen, Claire Baldwin, Maayken E. L. van den Berg, Isobel Harris, and Lucy K. Lewis in Journal of Aging and Health

Supplemental Material - Barriers and Facilitators to Physical Activity Among Older Adults in Residential Aged Care Facilities: A Mixed Methods Systematic Review Using the Social Ecological Model Supplemental Material for Barriers and Facilitators to Physical Activity Among Older Adults in Residential Aged Care Facilities: A Mixed Methods Systematic Review Using the Social Ecological Model by Sumana Baidya, Cath J. Evans, Jasmine M. Petersen, Claire Baldwin, Maayken E. L. van den Berg, Isobel Harris, and Lucy K. Lewis in Journal of Aging and Health
