# Supplementary material for: Barriers and Facilitators to Physical Activity Among Older Adults in Residential Aged Care Facilities: A Mixed Methods Systematic Review Using the Social Ecological Model
Source: J Aging Health. 2024 Nov 22;37(10):702–20. doi: 10.1177/08982643241302209 (PMC12541124; doi:10.1177/08982643241302209)
Supplement: Supplemental Material - Barriers and Facilitators to Physical Activity Among Older Adults in Residential Aged Care Facilities: A Mixed Methods Systematic Review Using the Social Ecological Model [file sj-pdf-2-jah-10.1177_08982643241302209.pdf]

ADDITIONAL FILE 2. SUMMARY OF INCLUDED STUDIES

Reference	Country	Study design	Setting (and brief description)	Participants	PA intervention	Data collection	Key findings	Methodological Quality
[1] Aro et al. (2018)	SAFR	Quant	RCF<br><br>These facilities offer independent living, assisted living and frail care living arrangements, providing increasing levels of assistance and nursing care	Residents: (n = 139)	NA	Questionnaires	Facilitators<br>• Knowledge of the benefits of regular PA, opportunities to socialise, encouragement by health care workers and availability of exercise facilities and trainers<br>Barriers<br>• Poor health status, lack of knowledge of the benefits of regular PA, lack of opportunities to socialise, lack of encouragement by health care workers and unavailable exercise facilities and trainers	87.5%
[2] Baert et al. (2016)	BELG	Mixed	LTCF<br><br>Institutional setting where 24-hour care is provided for older adult residents. The care provided includes onsite provision of personal assistance with activities of daily living, nursing and medical care	Staff - RACF administrators:<br>Qual: (n = 24)<br>Quant: (n = 127)	NA	Quant: surveys,<br>Qual: Semi-structured interviews	Facilitators<br>• Belief that WHO guidelines for PA are useful<br>• Integration into daily activities and education of LTCF staff regarding PA<br>Barriers<br>• Lack of staff, lack of adequate equipment, and lack of financial resources were rejected as potential barriers for organizing PA.	78%
[3] Baert et al. (2015)	BELG	Mixed	LTCF<br><br>Institutional setting where 24-hour care is	Staff – PT:<br>Qual: (n = 24)<br>Quant: (n = 254)	NA	Quant: surveys,<br>Qual: semi-structured interviews	Facilitators<br>• Motivated to organise PA to enhance the physical and psychological wellbeing of residents<br>• Social interaction (for residents) and	89%

			provided for older adult residents. The care provided includes onsite provision of personal assistance with activities of daily living, nursing and medical care				the positive effect of PA in reducing care burden are motivators<br>• PA is the basis of physiotherapeutic work and offering varied activities avoids PA becoming monotonous<br>Barriers<br>• Lack of time and the overload of paperwork<br>• Do not know the WHO PA guidelines for adults aged 65 and over, guidelines are useful, although may not be feasible for residents in LTCFs	
[4] Barrett et al. (2023)	IRELAND	Mixed methods (qualitative data)	NH<br><br>Facilities for older adults that are care-dependent (minimum capacity of 45 residents)	Staff (n=6) and residents (n=8)	Physical exercise program	Focus groups, semi-structure interviews	Facilitators (to program implementation)<br>• Leadership<br>• Motivation/ recognition of program importance<br>• Empowering and giving ownership to healthcare assistants<br>• Enjoyment (positive attitudes)<br>• Understanding of program (confidence to complete program, awareness of physical capabilities and limits)<br>• Perceived effectiveness of program (health benefits, structure & routine)<br>Barriers to program implementation<br>• Time pressures<br>• Staff shortages	80%
[5] Bender et al. (2021)	US	Qual	AL<br><br>Large (90+bed) community, with a separate 12-bed secure memory care unit	Residents: (n = 25)	NA	Interviews & observations	Facilitators & barriers<br>• The quality and location of the exercise program<br>• AL staffing limitations<br>• Residents' health and function<br>• Values about exercise<br>• Residents' interest in recreation and social engagement	70%
[6] Benjamin et al. (2009)	US	Qual	LTC	Staff - RACF administrators:	NA	Interviews	Facilitators	70%

			Facility that typically provides nursing care 24 hours a day, assistance with activities of daily living, room and board, and other services such as physiotherapy	(n = 9)			• Value of PA<br>Barriers<br>• Funding, human resources, and the built (physical) environment.<br>• The intersection of staffing issues and challenges in the built environment created less than optimal conditions for PA programs	
[7] Benjamin et al. (2011)	US	Qual	LTC<br><br>Facility that typically provides nursing care 24 hours a day, assistance with activities of daily living, room and board, and other services such as physiotherapy	Total: (n = 152)<br>Residents: (n=48)<br>Staff – PTs, health care aids, personal care attendants, PTs, managers: (n=62)<br>Family: (n=42)	NA	Focus groups	Facilitators<br>• All participants considered PA as important to health preservation.<br>Barriers<br>• Inadequate support for PA<br>• Pervasive institutional routines<br>• Physical environment constraints	80%
[8] Binns et al. (2023)	NZ	Qual	LTCF<br><br>Facilities for older adults that are no longer able to live independently at home	Staff – management and facilitators: (n=20)	Staying UpRight (SUp) falls prevention exercise program	Interviews and focus groups	Facilitators & barriers<br>• Business models and philosophies (financial constraints & no model of care to determine delivery of care)<br>• Requirements for evidence<br>• Valuing physical therapy for falls prevention	100%
[9] Bradwell et al. (2023)	UK	Qual	CH<br><br>CHs (1-bed to 83-beds), providing 24-h nursing care,	Key stakeholders (health and care staff): (n=44)	VR MOTUS Adventure omnidirectional treadmill	Focus groups & workshops	Facilitators<br>• Anticipated benefits (physical benefits; exercise, rehabilitation; wellbeing benefits: reducing boredom, social interaction,	80%

			residential care, or both for older adults who cannot remain at home				stimulates brain)<br>• Acceptability (positive comments)<br>Barriers<br>• Concerns of use (safety & secureness)<br>• Concerns of negative effects (unnatural movement, disorientating, fear)<br>• Suitability/ unsuitability (additional resourcing (staff), unsuitable for some)<br>• Improvements (safety, content)<br>• Current design (VR content)	
[10] Brett et al. (2023)	AUS	Quant	RACFs<br><br>Older adults who can no longer live in their own home. It includes accommodation and personal care 24 hours a day, as well as access to nursing and general health care services	Physiotherapists (n=165)		Survey	Facilitators & barriers<br>• Individual physiotherapist (work ethos and resilience, experience, knowledge)<br>• Collaborations (care coordination and working partnerships with different stakeholders)<br>• Organisational (resource (staffing/ available space/ equipment), procedural (administrative tasks and time))<br>• RACF community (lack of understanding of physios role, poor decision-making by RACF managers, culture among RACF stakeholders)<br>• Public policy (government policy, funding sources)	75%
[11] Brett et al. (2018)	AUS	Qual	NH<br><br>Facilities for older adults that are care-dependent	Total: (n = 19)<br>Staff – care: (n=10)<br>Family: (n=9)	Physical exercise intervention	Semi-structured interviews	Facilitators & barriers<br>• Influences of knowledge and understanding on individuals' views of physical exercise.<br>• Involvement of staff and family carers in research encourages their involvement in the implementation of research into clinical practice.	80%

[12] Brusco et al. (2024)	AUS	Mixed methods (qualitative data)	RACF<br><br>30-bed wing of a metropolitan residential aged care facility	Total n=31 Residents (n=26), allied health staff (n=5)	My Therapy Program	Survey & focus groups	Facilitators<br>• Simple drawing and explanations, family support, prompts for staff to complete program, framework for implementation, interprofessional collaboration, tailoring/ adaption for subpopulation (cognitive/ physical/ sensory impairments), integration into usual care<br>Barriers<br>• Remembering to complete program, difficult exercise, lack of motivation, visual impairment, scarce allied health resources, competing allied health staff demands, time required	80%
[13] Chao et al. (2016)	US	Qual	AL<br><br>One nonprofit 110-bed facility	Residents: (n=15)	Wii Fit exergaming program	Semi-structured interviews	Facilitators<br>• Health and mobility<br>• Increased alertness<br>• Elevated mindset<br>• Social interaction<br>• Structured program<br>Barriers<br>• Age or health related impairments to exercise<br>• Unpleasant experiences related to exercise	80%
[14] Chen and Li (2014)	TWAN	Qual	NH<br><br>Institution-based care for dependent people who need medical treatment or nursing care after discharge from a hospital or moving out of a	Residents: (n=18)	NA	Interviews	Facilitators<br>• Eagerness for returning home<br>• Fear of becoming dependent<br>• Improving mood state<br>• Filling empty time<br>• Previously cultivated habits	70%

			home					
[15] Chen (2010)	TWAN	Qual	LTC institution<br><br>Institution-based care for dependent people who need medical treatment or nursing care after discharge from a hospital or moving out of a home	Residents: (n = 90)	NA	Interviews	<u>Barriers</u><br>• Physical health problems and physical frailty<br>• Fear of resultant injury or falling<br>• Past sedentary lifestyle<br>• Insufficient understanding about PA<br>• Environmental restrictions.	80%
[16] Chu et al. (2021)	CAN	Mixed (qualitative data)	LTC home<br><br>Institutional setting that provides assistance with activities of daily living and primary medical care	Total: (n = 28)<br>Residents: (n=13)<br>Staff – support workers, managers, PT, PTA, life enhancement & activation therapist (n= 14)<br>Family: (n = 1)	Exergaming Intervention 'MouvMat'	Quant: outcome measures<br>Qual: Talk-aloud method & semi-structured interviews	<u>Facilitators</u><br>• Increase residents' autonomous PA.<br>• Improved physical and cognitive health	90%
[17] Chu et al. (2022)	CAN	Qual	LTC home<br><br>Nonprofit homes (128 and 350□bed facility)	Total: (n = 47)<br>Staff - care: (n = 21)<br>Family: (n=26)	Multifaceted walking intervention	Semi-structured interviews	<u>Facilitators</u><br>• Functional and physical improvements<br>• Behavioural and communication improvements<br>• Psychosocial improvements<br><u>Barriers</u><br>• Lack of time<br>• Opportunities for meaningful activity in LTC setting<br>• Staff experiences with resident	80%

							aggression	
[18] Galik et al. (2009)	US	Qual	NH<br><br>66-bed facility for older adults that are care-dependent	Staff- nursing assistants: (n = 7)	NA	Focus groups	Facilitators & barriers<br>• Knowing what makes them tick and move<br>• Teamwork and utilizing resources<br>• Barriers to restorative care.	80%
[19] Gebhard and Mir (2021)	AT	Qual	CH<br><br>Facilities accommodating on average n=72 residents	Residents (n =10)	NA	Semi-structured interviews	Facilitators<br>• Intrapersonal: ability to execute PA, enjoying PA, pastime and daily structure, PA in order to get from A to B, maintenance of former activities<br>• Interpersonal: group-based exercising with other residents, support for PA, PA as a joint activity with care giver, communication associated with PA<br>• Institutional/environment (NH): exercising outdoors, mobility aids<br>Barriers:<br>• Intrapersonal: physical, individual image on aging regarding oneself, psychological, cognitive<br>• Interpersonal: refusing group-based exercising with other residents, lack of support<br>• Institution/environment (NH), perception of missing opportunities for PA, constraints, feeling uneasy in the NH, design of the outdoor area, no appropriate music, mobility aids, rolling walkers<br>• Society: negative judgment regarding PA	100%
[20] Giné-Garriga et al. (2019)	SPN GRBR	Qual	CH<br><br>CHs are a long-	Total: (n= 70) University students: (n =	NA	Co-design workshops	Facilitators & barriers<br>• Perspectives about sedentary behaviour (SB)	90%

			term care setting where people live and have their care needs met in homely surroundings, usually for people needing more care than they could get in their own home or in supported housing	26)<br>Residents: (n = 22)<br>Staff - nurse, care assistants, PTs, OT, Geriatrician: (n = 14)<br>Family: (n=4)<br>Policymakers: (n = 4)			• Movement in care homes<br>• Assets for decreasing SB<br>• Strategies (e.g., undergraduate students involvement in co-creation process within a care home setting to raise their awareness, knowledge, skills and passion before entering the workforce	
[21] Gomaa et al. (2020)	AUS	Qual	RAC<br><br>60-bed high dependency residential aged care setting	Staff - dance instructor, class assistant, music therapist, PT, lifestyle program coordinator, RACF manager: (n= 7)	Music-cued dancing intervention	Semi-structured interviews	Facilitators<br>• Dance classes afforded motor and nonmotor benefits<br>• Support from management<br>• Resident supervision<br>• Age-appropriate music with a strong rhythmical beat<br>• Dance instructor skilled in comprehensive care<br>Barriers<br>• Multimorbidity<br>• Frailty<br>• Severe cognitive impairment<br>• Funding	100%
[22] Guerin et al. (2008)	AUS	Qual	RCF<br><br>Low-level residential care facility providing care for older people who need some help, but who do not have very complex ongoing care	Total: (n = 23)<br>Residents (n = 7)<br>staff – nurses, carers, PT, PTA, OT, OTA (n = 16)	NA	Focus groups	Facilitators<br>• Personal benefits, such as improved health and opportunities to socialise<br>• Support and encouragement that they received from family members and health professionals<br>Barriers<br>• Health issues (e.g., pain)<br>• Incontinence<br>• Hearing impairments<br>• External constraints (location of the	90%

			needs				classes and the early morning times)<br>• Lack of knowledge about the classes and the benefits of exercising<br>• Personal health status and systems-related motivators and barriers.	
[23] Högstedt (2023)	SWEDEN	Mixed methods (qualitative data)	LTCFs<br><br>Somatic and/or dementia care units of LTCFs (excludes short-term care, AL facilities, day care and community care)	Physiotherapists (n=97)		Survey	Facilitators<br>• Knowledge for active daily living (requires competences, establishing relationships, education)<br>• Adapt to present circumstances and priorities (PA needs to be prioritised, difficulties to adapt to limitations, plan according to resources and needs)<br>• Methods for exercise and to stay active in daily living (making the environment accessible and equipped for exercise and participation, diverse exercise types)<br>• Acts and adjustments for the resident (individualised physiotherapy measures, adjust to resident's interests<br>• Communication to promote activity (meetings for multi-disciplinary communication)	70%
[24] Hummer et al. (2015)	US	Mixed	AL<br><br>30-bed facility that provide a home-like residential program approach offering a range of care services to support resident independence	Total: (n=31)<br>Residents: (n=25)<br>Staff – nursing assistants (n= 6)	Sit-to-stand exercise program	Quant: Physical outcome measures<br>Qual: Surveys, surveys analysed for themes	Facilitators<br>• Nursing staff play an important role in breaking the cycle of dependence in residents, by actively engaging residents in daily exercise program<br>• Exercise program had an impact on residents' ADL function even with<br>Barriers<br>• Poor support of the program from both administration and the staff	45%
[25] Ingrid Brenner and	CAN	Quant	LTCF	Residents: (n=12)	NA	Physical outcome	Facilitators & barriers<br>• The amount of social support	75%

Marsella (2008)			100-bed facility for the elderly			measures, attendance rates, questionnaires	provided by significant others.<br>• the extent of chronic pain experienced by the client were the best predictors of engagement in regular exercise.	
[26] Jeon et al. (2019)	AUS	Qual	RAC home<br><br>For those who can no longer live in their own home. It includes accommodation and personal care 24 hours a day, as well as access to nursing and general health care services.	Residents & proxys where required: (n =24)	NA	Interviews	Facilitators & barriers<br>• Levels of activity prior to living in residential aged care<br>• Ageism<br>• Social capital and loss of a loved one<br>• Pain<br>• Staff support	90%
[27] Jepson et al. (2023)	UK	Mixed methods	Care homes<br><br>Institutions that provide 24-h nursing care, residential care, or both for older adults who cannot remain at home	Total n= 57 Residents living with dementia (n=15), managers (n=13), family members (n=7), care home staff members (n=22)		Survey and interviews, triangulation	Barriers<br>• Staff & cultural factors (division of labour, fixed routines, risk aversion)<br>• Characteristics of residents (physical abilities, lack of voice, mood, advanced dementia)<br>• Environment (space within care home setting, facilities within community)<br>• External factors (weather)<br>• Policies & practices (health & safety policies, risk management, actual resources)	56%
[28] Krafft et al. (2023)	GER	Mixed methods (qualitative data)	NH<br><br>Institution for those that are care dependent	Nursing home employees (n=14)	Individualized Cognitive and Physical Exercise-App (InCoPE-App)	Think aloud technique & survey	Facilitators<br>• Interface, user friendly<br>Barriers<br>• Navigation, screen layout (small font), graphics, comprehensibility (unclear terminology), time consuming, some features not intuitive, low digital	80%

							literacy	
[29] Kuk et al. (2018)	NETH	Quant	NH<br><br>Nursing home care is provided in somatic (care for those with physical problems) or psychogeriatric wards (e.g., care for those with dementia)	Staff – nurses, care assistants: (n = 368)	NA	Surveys	<u>Barriers</u><br>• Staffing levels<br>• Capabilities of residents<br>• Availability of resources<br>• Communication within the team<br>• A lack of referral to responsibilities<br>• Care routines	87.5%
[30] Lindelöf et al. (2012)	SE	Qual	RCF<br><br>The facilities comprise private apartments with access to common dining rooms, alarms, and on-site nursing and care	Residents: (n = 9)	High Intensity Functional Exercise program	Interviews	<u>Facilitators</u><br>• Belief in the positive effects of the programme, a strong desire to be active, and the will to strive to avoid further loss of capacity<br>• Physical and mental improvements that affected their daily life positively and that exercising in a group was stimulating and created a sense of togetherness.<br>• Support from the supervisors and belief in personal success<br><u>Barriers</u><br>• Struggling with failing bodies	90%
[31] Linhares et al. (2022)	BR	Qual	LTC institution<br><br>Private for-profit LTCs offering residential services and multi-disciplinary care (institutions accommodated 21-29 individuals)	Residents: (n = 9)	Recreational physiotherapy consisting of a multicomponent group exercise program	Semi-structured interviews	<u>Facilitators</u><br>• "activity for myself"<br>• "activity with others"<br>• "activity itself"<br>• Regular PA in recreational group, strengthens social bonds and provides physical and emotional well-being	80%

[32] Liu and Hu (2015)	CHIN	Quant	NH<br><br>Facility providing long-term functional support and nursing care for elders who require assistance with ADLs	Residents: (n = 34)	Educational program on PA	Questionnaires	Facilitators<br>• Educational intervention programs using different teaching strategies, such as discussions, demonstrations, and group exercises with low literacy materials<br>• Community health and nursing home nurses must take responsibility for providing PA education	37.5%
[33] Lu et al. (2011)	US	Qual	AL facility<br><br>Facilities had ≥10 beds and on average 44-120 units/ apartments	Residents: (n=50)	NA	Focus groups	Facilitators<br>• Safety<br>• Comfort/convenience<br>• Aesthetics<br>• Continuity and grasp-ability of handrails<br>• Coverage of carpeted floor<br>• Availability of seating<br>• Appropriate size of the corridor (i.e., width and length) and the elevator<br>• Appropriate locations of activity spaces and restrooms<br>• Presence of artwork, window views, and plants	70%
[34] Mahrs Träff et al. (2020)	SE	Qual	AL facility<br><br>Facilities wherein individuals rent their apartment with their own lease	Total: (n = 30)<br>Residents: (n = 13)<br>Staff – PT, OT, care professionals, head of units (n= 17)	NA	Interviews & observation	Facilitators & barriers<br>• Physical environment influences the opportunities for PA<br>• An unsuitable environment limits the ability of older people to engage in PA	90%
[35] Mahrs Träff et al. (2019)	SE	Qual	AL facility<br><br>Facilities wherein individuals rent their apartment	Total: (n= 17)<br>Residents: (n = 13)<br>Staff – PT: (n = 4)	NA	Interviews & observation	Facilitators and barriers<br>• PA was not a focus in the assisted living facilities, and recommendations on PA were not followed	80%

			with their own lease				• Those in need of assistance had limited possibilities to be active<br>• There was a need for PA that the staff do not necessarily and sufficiently identify<br>• Variations in how older people engaged in PA and how PA were part of everyday life<br>• Physiotherapists played no clear role at the facilities, especially with regard to preventive exercise<br>• Older individuals were not involved in determining which activities should be made available to the residents	
[36] Meyer et al. (2023a)	AUS	Mixed methods (qualitative data)	LTC<br><br>Home provides high-level care for up to 126 residents, including a memory support unit	Older veterans with dementia and family (or nominated representative) Care home staff, management and volunteers (n=9)	Weaving Evidence into Action for Veterans with Dementia (WEAVE)	Survey, interviews (with staff)	<u>Facilitators</u><br>• Therapist-led sessions (with assistance critical and whole of care home input)<br>• Coordinated programming to maximise attendance<br>• Identification of funding sources for sustainability and programme expansion<br>• Flexible scheduling to accommodate morning and afternoon preferences<br>• Dedicated physical space and appropriate equipment<br>• Visibility and positive promotion of the programme among staff, residents and families<br>• A trained and enthusiastic group of leaders to co-ordinate and support the programme across the care home<br>• Bringing staff/ volunteers on the journey through experiential learning about programme component, empowering their ability to continue the activities post session	50%

[37] Meyer et al. (2023b)	AUS	Qual	Care homes<br><br>Home provides high-level care for up to 126 residents, including a memory support unit.	Veterans (with dementia; n=11) and family members (n=1) Care home LTC staff and volunteers (n=17)	Weaving Evidence into Action for Veterans with Dementia (WEAVE)	Focus groups	Facilitators<br>• Outcomes/ motivations (physical & mental health benefits)<br>• Credentialled/ qualified leaders<br>• Effective scheduling of activities<br>• Champions to generate enthusiasm and positivity<br>Barriers<br>• Reasons for avoiding program (health, fear)	80%
[38] Mihalko and Wickley (2003)	US	Quant	AL facility<br><br>Facilities provide a home-like residential program approach offering a range of care services to support resident independence (≥ 15 residents)	Staff – executives directors of AL Facilities<br>n = 21	NA	Interview & observation	Facilitators<br>• Successful interventions for the promotion of lifestyle behaviour change should include multiple perspectives about PA including older adults, facilitating agents within the residence (i.e., administrators, staff, and physicians, as well as family members), and interventionists and researchers	75%
[39] Moran (2015)	AUS	Mixed (qualitative data)	AL	Residents<br>Quant: (n= 20)<br>Qual (n= 13)	NA	Quant: physical outcome measures<br>Qual: focus groups	Facilitators<br>• Social and physical environments<br>• Social support<br>• Staff involvement<br>Barriers<br>• Health concerns, fear of injury and motivation<br>• Perceived difficulty of PA and old age	70%
[40] Narkauskaitė-Nedzinskienė et al. (2020)	LT	Mixed (qualitative data)	Social care home<br><br>Care institution with either a strictly structured environment (partially	Total: (n = 40)<br>PI: (n=20)<br>FI: (n=20)	FI: Control group<br>PI: Adapted Physical Activity program	Quant: physical outcome measures<br>Qual: semi-structured Interviews	Facilitators & barriers<br>• Self-expression<br>• Self-awareness<br>• Body image<br>• Self-esteem	80%

			independent persons; PI) or inclusive environment (fully independent persons; FI)					
[41] Narsakka et al. (2023)	FINLAND	Qual	LTC<br><br>Large buildings with several smaller units (older adults pay rent for rooms) that have common areas for dining and activities	Residents (n=10), staff members (n=12)		Photo-elicitation	<u>Facilitators and barriers</u><br>• Facilities should be designed and equipped for users (designs and aids for mobility (spacious rooms), physical environment in support of outdoor activity)<br>• Moving in the institutional environment (restricted freedom of movement, social environment)<br>• Passivity as a norm (PA sparse; inactivities of daily life, facilities and equipment for exercise but no one to facilitate it)<br>• Nurses daily practice and resources (nurses require new kind of thinking, lack of staff/ time)	100%
[42] Ofosu et al. (2023)	SCOTLAND	Mixed methods (qualitative data)	Care homes<br><br>Facilities that provide older adults with residential, nursing, Alzheimer and dementia care	Residents (n=49), activity coordinators (n=5)	Digital movement and music program	Focus groups, semi-structured interviews and questionnaire	<u>Facilitators</u><br>• Progressive and future modifications (integration into care home schedule, encouragement)<br>• Improved mood and physical health<br>• Increased job satisfaction (care home staff)<br>• Social support (positive resident-resident and resident-staff relationships)<br><u>Barriers</u><br>• Motivation and engagement (lack of motivation)<br>• Health conditions (cognitive impairment and disabilities)	80%

							• Death & hospitalisation<br>• Resources for delivery (lack of resources; staff shortage and absence, technical problems, limited space)	
[43] Olsen et al. (2015)	NO	Qual	NH<br><br>Institution-based 24-hour care for dependent people who need medical treatment or nursing care that cannot be provided in the home	Residents (n = 8)	High-Intensity Functional Exercise (HIFE) program	Semi-structured interviews	Facilitators<br>• Pushing the limits<br>• Being invested<br>• Relationships facilitate exercise participation<br>• Exercise revives the body, increases independence, and improves self-esteem<br>• PA is a basic human necessity-use it or lose it!	90%
[44] Phillips and Flesner (2013)	US	Qual	AL + RC<br><br>Facilities ranged in capacity from 30-87 occupants, catering to those primarily requiring personal-care services	Residents (n = 47)	NA	Focus groups	Facilitators & barriers<br>• Walking indoors and out<br>• Performing professionally prescribed home exercises<br>• Using available exercise equipment<br>• Past PA experiences shaped current preferences and practices.<br>• Value of PA<br>• Strategies to facilitate PA<br>• Support needs to promote PA.<br>• Lack of dedicated exercise space and short corridors hampered efforts to stay active<br>• Individualized home exercise programs and supervised exercise sessions	100%
[45] Pienaar et al. (2004)	SAFR	Quant	Old age home<br><br>A LTCF that provide accommodation	Residents (n= 390)	NA	Questionnaires	Facilitators<br>• General knowledge of the influence PA on life quality<br>• Most of the participants (80.5%)	87.5%

			for older adults and support residents with daily living activities, and additional specialised care where required				enjoyed exercising and 60% had a positive attitude towards exercise.<br>Barriers<br>• Most participants (62.8%) felt that they had not received enough information about PA from their doctor	
[46] Post et al. (2020)	AUS	Qual	RACF<br><br>Facilities that provide accommodation for older adults and support residents with daily living activities, and additional specialised care where required	Total: (n = 24)<br>staff – care: (n=15)<br>Family: (n=9)	EP delivered exercise program	Interviews	Facilitators & barriers<br>• Perceptions prior to and after the programme<br>• Benefits associated with participation in programme<br>• Acceptability of the programme insofar as its impact on the roles of care staff<br>• Barriers to implementation	70%
[47] Poveda-López et al. (2023)	SPN	Qual	LTC<br><br>Institutional settings that provide a range of services (e.g., assistance with activities of daily living) for older adults with reduced cognitive or physical function	Residents (n=47)		Focus groups	Facilitators & barriers<br>• Perceptions about health professions delivering the exercise programs (professionalism, availability, treatment (infantilise)<br>• Facilities (lack of space, resources, lack of staff members)<br>• Organization (functional status)	90%
[48] Poveda-López et al.	SPN	Qual	LTC	Residents: (n=36)	NA	Focus groups, modified	Facilitators	90%

(2022)			Institutional settings that provide a range of services (e.g., assistance with activities of daily living) for older adults with reduced cognitive or physical function			grounded theory	• Attitudes and motivations towards the exercise programs<br>• Self-perceived health<br>• Knowledge of the concept of PA<br>• Perceived effects of the exercise programs<br>• Wishes or expectations for the exercise programs (increase frequency of exercise sessions, to exercise outdoor and to increase walking times)	
[49] Prevc and Topič (2009)	SVN	Quant	NH<br><br>NHs provide institutionalised forms of care for the elderly (who can no longer live at home) including social nursing services	Residents: (n=75)	NA	Questionnaires	<u>Facilitators</u><br>• Interaction (socialising) with other residents an important component of PA	50%
[50] Rapp and Carlson (1987)	US	Quant	NH<br><br>Facility for older adults that are care-dependent (120-bed care unit)	Total: (n= 106)<br>Residents: (n= 67)<br>Staff – nurses & nursing aids: (n= 39)	NA	Questionnaires	<u>Facilitators</u><br>• 56% of the residents perceived it is important to increase their level of exercise and 63% said they would participate in such programs<br>• Improve quality of life, physical health and mood<br>• Staff members perceived that an increase in exercise by residents would be feasible, beneficial, important, and would require frequent attention from staff.<br><u>Barriers</u><br>• Both staff and residents perceived that	25%

							an increase in exercise may be difficult to achieve and would meet with some disinterest.<br>• Belief that exercise periods had to be strenuous and long to be effective	
[51] Raynor et al. (2020)	AUS	Mixed (qualitative data)	RCF<br><br>Low-level residential care facility providing care for older people who need some help, but who do not have very complex ongoing care needs	Quant: residents: (n=23)<br>Qual: total (n = 18)<br>Residents: (n=5)<br>Family (n=3)<br>Staff – care (n=6)<br>Volunteer (n=1)<br>Research team members: (n=3)	1:1 exercise program involving a components-approach intervention	Quant: Physical outcome measures<br>Qual: Interviews	<u>Facilitators</u><br>• Individualized structure of the AEP program, which was considered integral to participants' enjoyment and progress.<br>• One-on-one sessions tailored to individual needs provided a number of benefits, including the opportunity to foster personal connections, and accommodate specific needs relating to cognitive and sensory impairments.	80%
[52] Resnick et al. (2008)	US	Qual	NH<br><br>Facilities that provide care to older adults that are medically, functionally, and cognitively impaired	Staff – nursing assistants (n=93)	The Res-Care Intervention is a 2-tiered intervention focused on motivating NAs to engage in restorative care activities and teaching them how to motivate the residents to do likewise.	Focus groups	<u>Facilitators & barriers</u><br>• Facilitators of restorative care<br>• Barriers to restorative care<br>• Benefits of restorative care<br>• Sustaining restorative care	60%
[53] Rohisha et al. (2017)	IN	Quant	Old age home<br><br>These homes provide accommodation and a spectrum of health, personal,	Residents: (n=29)	NA	Self-administered questionnaire	<u>Facilitators</u><br>• Improve mental health (93.1%) and reduced stress (86.2%)<br>• Perceived self-efficacy for performing exercise (feeling confidence; 79.3%), and self-worthy (82.8%) to perform exercise	37.5%

			and supportive services to the elderly who are unable to be cared for at home				• Motivation by the care givers (93.1%) to perform exercise and lack of awareness of the benefits of exercise from media (65.5%)<br>Barriers<br>• Difficulty performing PA in old age (69%) and lack of information (55.2%)<br>• Lack of facilities to perform exercise, and no supervisor or trainer for exercise	
[54] Ruuskanen and Parkatti (1994)	FIN	Quant	NH<br><br>Facilities that provide 24-hour care to older adults that are medically, functionally, and/or cognitively impaired	Residents: (n=143)	NA	Structured interview (descriptive stats)	Facilitators<br>• PA was associated with self-rated functioning, depressive symptoms, and contentment with life<br>Barriers<br>• Poor health status	75%
[55] Saravanakumar et al. (2018)	AUS	Qual	RAC<br><br>108-bedded facility offering low- and high-dependency residential care for older persons	Total: (n = 19)<br>Residents: (n=16)<br>Staff – recreational activity officers (n=3)	Tai-chi & Yoga program	Focus groups	Facilitators<br>• Novel, new and exciting<br>• Smoothness, rhythm and flow<br>• Slow and mindful<br>• Gentle but rewarding<br>• Moving whole body<br>• Perceived benefits<br>• Worthwhile<br>• Feeling alive<br>• Calming and relaxing<br>• Enhance quality of life through interaction of physical, emotional and intellectual wellness domains	90%
[56] Stathi and Simey (2007)	GRBR	Qual	NH	Residents Pre-	Exercise intervention	Semi-structured	Facilitators<br>• Improved quality of life through better	70%

			Facilities that provide 24-hour care to older adults that have complex health care needs	intervention: (n= 14)<br>post-intervention: (n= 7)	delivered by a postural-stability instructor	interviews	mobility, decreased fear of falling, and feelings of achievement and success<br>• Better functioning and mobility, more independence, and more social interaction and fun<br>• Valued program as an opportunity to do something for themselves, to add something to their weekly routine, to meet other people, and to be more active generally<br>• Professionalism of the exercise instructor, balancing principles of safe and effective practice with the need to ensure that participants had fun in a supportive environment	
[57] Swales et al. (2024)	UK	Mixed methods (qualitative data)	RACF<br><br>Facility offering residential, nursing, and dementia care in one setting, allowing older adults with varying care needs to live together in a supportive community	Residents (n=11)	Progressive resistance training	Semi-structured interviews, focus groups	<u>Facilitators</u><br>• Implementation (session time flexibility)<br>• Practicality (equipment suitability and accessibility)<br>• Integration (fit into existing culture)<br>• Expansion (enthusiastic person/ consistent contact to run intervention)	80%
[58] Taylor et al. (2020)	US	Qual	LTCF<br><br>LTCFs include nursing homes, skilled nursing facilities, and assisted living facilities	Residents (n = 4)	'Bingocize' program, combining falls prevention exercises with bingo	Semi-structured interviews	<u>Facilitators</u><br>• Attitude<br>• Perceived norms<br>• Personal agency (self-efficacy & perceived control)<br>• Intergenerational connection<br>• Enjoyment	80%

[59] Turpie et al. (2017)	GRBR	Qual	CH<br><br>Setting in which resident's needs can be met by trained staff	Staff -care & management: (n = 8)	'Let's Motivate' a training intervention aimed at inspiring staff to get residents to move more often	Semi-structured interviews	<u>Facilitators</u><br>• Practical demonstrations<br>• Simplicity of training<br>• Rationale for training<br>• Appropriate environments for PA (e.g., space_<br>• Access to equipment<br>• Supported by staff<br>• Integration into daily activities<br><u>Barriers</u><br>• Physical health (mobility)<br>• Reduced cognitive status (e.g., dementia)<br>• Willingness to participate<br>• Fear<br>• Resistance from staff<br>• Lack of staff	80%
[60] Vos et al. (2019)	US	Qual	AL<br><br>Facilities had a combined capacity of 176 residents, were all one-story buildings with a central dining room, and provided a range of services (e.g., assistance with activities of daily living as needed).	Residents (n = 29)	NA	Semi-structured interviews	<u>Facilitators & barriers</u><br>• PA was experienced as planned exercise, activities of daily living, and social activities based on a schedule or routine<br>• PA meant independence and confidence in the future<br>• Residents perceived themselves as being physically active<br>• Social comparisons influenced perception of PA<br>• Personal health influenced PA<br>• Motivations and preferences influenced PA	90%
[61] Wang et al. (2021)	CHIN	Qual	NH	Residents (n =13)	Chinese square-dancing	Semi-structured	<u>Facilitators</u><br>• Benefits associated with exercise	70%

			Facility providing long-term functional support and nursing care for elders who require assistance with ADLs		program	interviews	(physical health, spiritual and psychological health, social interaction, cognition, health literacy)<br>• Facilitators of adherence (positive experiences, staff related factors (supervision and guidance, understanding and support for staff))<br><u>Barriers</u><br>• Individual factors (physical discomfort, forgetting and competing priorities)<br>• Activity factors (duration of PA too long)	
[62] Webster et al. (2023)	US	Qual	AL facility<br><br>Facilities provide 24-hour supportive care (including meals, personal care, housekeeping, and assistance with activities of daily living), primarily for older adults who have functional limitations but do not need the level of medical supervision provided by nursing homes	Residents (n=20)	NA	Semi-structured interviews	<u>Facilitators & barriers</u><br>• Time period (shorter one-hour sessions)<br>• Framing the intervention as increasing light PA rather than decreasing SB<br>• Motivation to be active<br>• Safety concerns<br>• Ageist attitudes about PA<br>• Varying abilities of residents<br>• Social influences<br>• Limited opportunities for PA	70%

[63] Weeks et al. (2008)	CAN	Qual	LTCF<br><br>Privately operated, 112-bed skilled nursing home	Residents: (n=7)<br>**Data not extracted for community dwelling participants (n=17)*	NA	Semi-structured interviews	<u>Facilitators and barriers</u><br>• Past experiences, life transitions, and future concerns influence seniors' participation in PA<br>• Intergenerational influences<br>• Establishment of early PA patterns<br>• Family transitions over the life course<br>• Changing health status over the life course<br>• Future health concerns.	60%
[64] Weissbach et al. (2023)	GERMANY	Mixed methods	NH<br><br>Facilities that provide 24-hour supportive care for older adults often characterised by multi-morbidity and frailty, and have difficulty with activities of daily living	Qual; Residents (n=11), volunteers (n=12)<br>Quant; volunteers (n=40)	Prevention by lay-assisted Outdoor-Walking in the Elderly at Risk (POWER)	Semi-structured interviews, focus groups, survey (triangulated)	<u>Facilitators</u><br>• Motivation for participation (interest in PA)<br>• Self-perceived physical and mental effects<br>• Experiences during intervention (increased safety, mutual benefits (volunteer & resident), fluctuating motivation according to health conditions)<br>• Project organization (random allocation of volunteers and residents different ages and genders, social support/ encouragement)<br><u>Barriers</u><br>• Perceived challenges (environment (weather, suitability of NH for walks), poor health, lack of motivation)	83%
[65] Wootten et al. (2022)	AUS	Qual	RCF<br><br>Low-level residential care facility providing	Residents: (n=7)	NA	Interviews	<u>Facilitators</u><br>• Positive socialisation (inclusion) with subthemes of enjoying social contact and avoiding "zombieland" (loneliness)	80%

			care for older people who need some help, but who do not have very complex ongoing care needs				• Maintaining independence was the benefit, with subthemes of getting around to socialise, activities of daily living and preserving health to socialise.<br>Barriers<br>• Negative socialisation (isolation) with subthemes of avoiding conflict and feeling awkward.	
[66] Wu et al. (2013)	TWAN	Qual	LTCF<br><br>Institution-based care for dependent people who need medical treatment or nursing care after discharge from a hospital or moving out of a home.	Staff- nurses: (n=20)	NA	Semi-structured interviews	Facilitators<br>• Recognising the importance of participation in PA<br>• Encouraging participation in PA<br>• Respecting the autonomy of the residents<br>• Preventing falls<br>• Facing a dilemma	80%
[67] Yokogawa et al. (2023)	JAPAN	Qual	LTCF<br><br>Facility for older people to reside that offers health and/or personal care services	Physiotherapists (n=4)	Physiotherapy session (included exercise program and gait training)	Semi-structured interviews	Facilitators<br>• Make structured preparations for clients to being PA (understand client's condition, maintain familiar movement patterns)<br>• Link exercise therapy to a client's daily life (convey information in living environment, markers for self-pacing, non-verbal reminders)<br>• Discover changes in daily life (identify activities client is naturally good at, ascertain recent lifestyle habits, notice changes in life function)<br>• Ascertain cognitive function<br>• Accommodate client differences (set goals considering changes in cognitive function, set individual	80%

								standards for evaluating the degree of independence in ADL)	

AUS = Australia, AT = Austria, BELG = Belgium, BR = Brazil, CAN= Canada, CHIN = China, FIN = Finland, GRBR= United Kingdom, IN = India, SAFR = South Africa, TWAN = Taiwan, US = United States of America, NETH = Netherlands, SVN = Slovenia, SPN = Spain, NO = Norway, LT = Lithuania, SE = Sweden

LTCF = Long term care facility, RACF = Residential aged care facility, RC= Residential care, AL = Assisted living, NH = Nursing home, CH = care home, OAH = old aged home, PI = Social Care Home for Elderly People, IL = Independent living

EP = Exercise Physiologist, PT = Physiotherapist, PTA = Physiotherapy Assistant, OT = Occupational Therapist, OTA Occupational Therapy Assistant, SDM: Substitute-decision-makers

PA = Physical activity, NA = Not applicable
