# Supplementary material for: Barriers and Facilitators to Physical Activity Among Older Adults in Residential Aged Care Facilities: A Mixed Methods Systematic Review Using the Social Ecological Model
Source: J Aging Health. 2024 Nov 22;37(10):702–20. doi: 10.1177/08982643241302209 (PMC12541124; doi:10.1177/08982643241302209)
Supplement: Supplemental Material - Barriers and Facilitators to Physical Activity Among Older Adults in Residential Aged Care Facilities: A Mixed Methods Systematic Review Using the Social Ecological Model [file sj-pdf-3-jah-10.1177_08982643241302209.pdf]

ADDITONAL FILE 3. EXAMPLES OF QUALITISED DATA.

Study	Data collection and analysis method/s	Quantitative data	Qualitised data	Final themes and sub-themes	SEM
[53] Rohisha et al. 2017	Self-administered questionnaires, descriptive analyses	**Barriers**			
		I believe performing exercise:<br>- Will cause pain on body (41.4%)<br>- Will not bring long term benefits (31%)<br>- Is difficult due to loss of strength in old age (69%)<br>- I lack information regarding the exercise (55.2%)	Exercise causes pain<br>Perception of no long-term benefit<br>Exercise is difficult due to lack of strength in old age<br>Lack of information about exercise	Negative attitudes<br>- negative beliefs<br><br>Limitations<br>- sensory limitations<br>- physical limitations<br><br>Lack or, or poor information sharing	Intra-personal<br><br><br><br><br><br><br>Organisational
		**Facilitators**			
		I feel performing exercise:<br>- Will make the body physically healthy (89.7%)<br>- Will improve mental health (93.1%)<br>- Will reduce stress (86.2%)<br>- Will maintain BP in normal limits (58.6%)<br>- Will good sleep at night (82.8%)<br>- Will make me look better (79.3%)	Improved physical health,<br>Improved mental health<br>Reduced stress<br>Improved sleep<br>Maintained normal blood pressure<br>Improved sleep<br>Improved appearance<br>Happy feelings<br>Increased energy<br>Enjoyment<br>Caregiver motivation<br>Motivation through watching	Positive feelings<br>-promotes enjoyment, fun and happiness<br><br>Self-awareness<br>-improves self-image and esteem<br><br>Benefits of PA<br>- general health and wellbeing benefits<br>- physical health benefits<br>- physiological benefits	Intra-personal

		Activity related affect:<br>Performing exercise makes me happy (89.7%), sad (0%), energetic (65.5%), bored (6.9%), my daily life more enjoyable (65.5%)<br>93.1% of participants were motivated by caregivers to perform the exercise<br>62.1% encouraged by seeing their friends perform the exercise<br><br>Interpersonal influences:<br>- Care are givers motivate me to perform exercise (93.1%)<br>- My friends influence me to perform exercise, I feel encouraged when I see my friends performing exercise (62.1%)	peer (friend) involvement in exercise	- psychological benefits<br><br>Support and encouragement<br>- support and encouragement of residents	 <br>Inter-personal
